# Supplementary material for: Performance Comparison of Four Hepatitis E Antibodies Detection Methods
Source: Microorganisms. 2024 Sep 11;12(9):1875. doi: 10.3390/microorganisms12091875 (PMC11434459; doi:10.3390/microorganisms12091875)
Supplement: Supplementary file 1 [file microorganisms-12-01875-s001.zip › microorganisms-3117454-supplementary.pdf]

Table S1. Raw data of anti-HEV IgM panel I and II samples in four used techniques (LIAISON, VIRCLIA, WANTAI and VIDAS). POS: positive result, NEG: negative result, IND: indeterminate result, IS: insufficient sample.

Panel	Sample ID	LIAISON		VIRCLIA		WANTAI		VIDAS	
		Label	Index	Label	Index	Label	Index	Label	Index
IgM+RNA (I)	P1-001	POS	5.7	POS	14.256	POS	8.88	POS	34.43
IgM+RNA (I)	P1-002	POS	3.01	POS	9.458	POS	9.02	POS	7.98
IgM+RNA (I)	P1-003	POS	5.15	POS	10.899	POS	9.35	POS	16.03
IgM+RNA (I)	P1-004	POS	5.99	POS	10.531	POS	9.55	POS	30.48
IgM+RNA (I)	P1-005	POS	5.09	POS	6.045	POS	9.06	POS	26.62
IgM+RNA (I)	P1-006	POS	4.2	POS	24.626	POS	9.73	POS	11.35
IgM+RNA (I)	P1-007	POS	5.5	POS	11.512	POS	9.91	POS	19.18
IgM+RNA (I)	P1-008	POS	4.82	POS	10.414	POS	10.06	POS	19.31
IgM+RNA (I)	P1-009	POS	5.52	POS	11.309	POS	9.05	POS	41.98
IgM+RNA (I)	P1-010	POS	5.68	POS	23.529	POS	9.25	POS	38.03
IgM+RNA (I)	P1-011	POS	4.29	POS	4.151	POS	9.12	POS	9.51
IgM+RNA (I)	P1-012	POS	4.18	IND	0.423	POS	2.61	POS	8.03
IgM+RNA (I)	P1-013	POS	3.86	POS	11.375	POS	9.22	POS	2.26
IgM+RNA (I)	P1-014	POS	3.54	IS	IS	POS	9.67	POS	10.31
IgM+RNA (I)	P1-015	POS	5.76	POS	6.107	POS	10.08	POS	40.13
IgM+RNA (I)	P1-016	POS	5.18	POS	11.992	POS	9.47	POS	28.42
IgM+RNA (I)	P1-017	POS	5.68	POS	24.489	POS	8.91	POS	36.75
IgM+RNA (I)	P1-018	POS	5.36	POS	19.827	POS	9.30	POS	39.32
IgM+RNA (I)	P1-019	POS	4.8	POS	9.553	POS	9.29	POS	19.38
IgM+RNA (I)	P1-020	POS	3.89	POS	25.848	POS	9.37	POS	17.86
IgM+RNA (I)	P1-021	POS	5.77	POS	11.85	POS	5.42	POS	25.4
IgM POS (II)	P2-001	POS	2.77	NEG	0.151	IND	1.04	NEG	0.84
IgM POS (II)	P2-002	POS	3.84	POS	4.678	POS	8.75	POS	8.37
IgM POS (II)	P2-003	POS	3.76	NEG	0.159	NEG	0.34	POS	8.3
IgM POS (II)	P2-004	POS	4.5	POS	4.5	POS	6.47	POS	10.95
IgM POS (II)	P2-005	POS	5.69	POS	14.861	POS	9.38	POS	37.32
IgM POS (II)	P2-006	POS	2.59	POS	1.417	POS	6.34	POS	6.67
IgM POS (II)	P2-007	POS	5.18	POS	0.482	NEG	0.67	POS	20.1
IgM POS (II)	P2-008	POS	3.66	POS	7.559	POS	8.89	POS	8.7
IgM POS (II)	P2-009	POS	1.12	POS	0.605	POS	1.32	NEG	0.46
IgM POS (II)	P2-010	POS	1.48	POS	12.903	POS	9.97	POS	3.17
IgM POS (II)	P2-011	POS	4.27	POS	4.632	POS	8.44	POS	4.32
IgM POS (II)	P2-012	POS	4.25	POS	7.478	POS	8.64	POS	11.34
IgM POS (II)	P2-013	POS	5.78	POS	19.192	POS	9.10	POS	39.55
IgM POS (II)	P2-014	POS	4.72	POS	14.539	POS	8.73	POS	16.5
IgM POS (II)	P2-015	POS	1.11	NEG	0.038	NEG	0.01	NEG	0.68
IgM POS (II)	P2-016	POS	2.54	POS	0.816	POS	3.61	POS	3.29
IgM POS (II)	P2-017	POS	4.48	POS	11.216	POS	9.72	POS	14.69
IgM POS (II)	P2-018	POS	5.16	POS	14.565	POS	8.89	POS	19.13
IgM POS (II)	P2-019	POS	4.25	POS	6.582	POS	8.88	POS	11.52

IgM POS (II)	P2-020	POS	6.09	POS	14.358	POS	8.54	POS	11.71
IgM POS (II)	P2-021	NEG	0.935	POS	0.556	POS	1.25	NEG	0.89
IgM POS (II)	P2-022	POS	3.22	POS	1.569	POS	3.27	POS	3.09
IgM POS (II)	P2-023	POS	5.41	POS	23.608	POS	10.13	POS	28.51
IgM POS (II)	P2-024	POS	4.54	POS	2.067	POS	6.36	POS	8.56
IgM POS (II)	P2-025	POS	5.11	POS	16.4999	POS	8.82	POS	19.19
IgM POS (II)	P2-026	POS	6.72	POS	21.097	POS	9.24	POS	38.65
IgM POS (II)	P2-027	POS	5.55	POS	8.7	POS	9.32	POS	15.95
IgM POS (II)	P2-028	POS	3.13	POS	7.392	POS	9.70	POS	6.04
IgM POS (II)	P2-029	POS	1.64	POS	6.063	POS	9.07	POS	3.9
IgM POS (II)	P2-030	POS	5.57	POS	13.233	POS	9.05	POS	32.8
IgM POS (II)	P2-031	POS	3.24	NEG	0.105	NEG	0.23	POS	4.48
IgM POS (II)	P2-032	POS	1.43	POS	1.298	POS	4.90	POS	2.54
IgM POS (II)	P2-033	POS	5.29	POS	28.336	POS	8.05	POS	35.11
IgM POS (II)	P2-034	POS	4.55	POS	13.307	POS	9.03	POS	26.2
IgM POS (II)	P2-035	POS	5.59	POS	14.112	POS	9.45	POS	36.94
IgM POS (II)	P2-036	POS	3.79	POS	15.729	POS	10.44	POS	15.43
IgM POS (II)	P2-037	POS	1.22	POS	1.598	POS	6.97	POS	2.99
IgM POS (II)	P2-038	POS	5.77	POS	11.248	POS	9.69	POS	38.65
IgM POS (II)	P2-039	POS	1.63	IS	IS	POS	1.46	NEG	0.58
IgM POS (II)	P2-040	NEG	0.479	NEG	0.047	NEG	0.01	NEG	0.11
IgM POS (II)	P2-041	POS	4.64	IS	IS	POS	8.92	POS	36.65
IgM POS (II)	P2-042	POS	3.83	POS	7.705	POS	9.50	POS	24.77
IgM POS (II)	P2-043	POS	3.8	POS	0.755	POS	3.99	POS	7.82
IgM POS (II)	P2-044	NEG	0.544	NEG	0.211	POS	1.49	NEG	0.61
IgM POS (II)	P2-045	POS	2.09	NEG	0.117	POS	1.63	POS	4.83
IgM POS (II)	P2-046	POS	1.03	POS	0.523	NEG	0.73	POS	2.26
IgM POS (II)	P2-047	POS	6.29	POS	10.281	POS	10.28	POS	20.12
IgM POS (II)	P2-048	POS	4.49	NEG	0.106	NEG	0.09	POS	8.26
IgM POS (II)	P2-049	POS	3.5	NEG	0.074	NEG	0.05	POS	3.6
IgM POS (II)	P2-050	POS	1.91	POS	4.397	POS	5.82	POS	3.73
IgM POS (II)	P2-051	POS	1.78	POS	0.914	POS	2.06	POS	2.57
IgM POS (II)	P2-052	POS	1.73	POS	12.264	POS	11.46	POS	3.34
IgM POS (II)	P2-053	POS	3.22	NEG	0.076	NEG	0.27	NEG	0.97
IgM POS (II)	P2-054	POS	4.89	POS	3.185	POS	5.11	POS	7.88
IgM POS (II)	P2-055	POS	0.863	NEG	0.124	NEG	0.20	NEG	0.8
IgM POS (II)	P2-056	POS	4.59	POS	18.067	POS	11.74	POS	11.77
IgM POS (II)	P2-057	POS	1.27	NEG	0.382	NEG	0.40	NEG	0.96
IgM POS (II)	P2-058	POS	3.17	NEG	0.262	POS	1.12	POS	3.25
IgM POS (II)	P2-059	POS	3.31	POS	1.756	POS	3.65	POS	4.26
IgM POS (II)	P2-060	POS	5.38	POS	2.673	POS	2.57	POS	9.2
IgM POS (II)	P2-061	POS	3.89	NEG	0.044	NEG	0.07	POS	3.99
IgM POS (II)	P2-062	POS	1.5	POS	0.878	POS	2.95	POS	1.91
IgM POS (II)	P2-063	POS	5.63	POS	6.142	POS	8.56	POS	10.92
IgM POS (II)	P2-064	POS	3.46	POS	24.587	POS	11.98	POS	11.21
IgM POS (II)	P2-065	POS	6.21	POS	20.142	POS	11.64	POS	36.42

IgM POS (II)	P2-066	POS	6.88	POS	23.279	POS	13.29	POS	39.19
IgM POS (II)	P2-067	POS	6.33	NEG	0.293	NEG	0.33	POS	20.17
IgM POS (II)	P2-068	POS	5.92	POS	2.563	POS	7.80	POS	27.88
IgM POS (II)	P2-069	POS	7.22	POS	3.462	POS	8.08	POS	14.27
IgM POS (II)	P2-070	POS	1.32	IND	0.458	NEG	0.48	NEG	0.56
IgM POS (II)	P2-071	POS	1.67	POS	0.56	IND	0.98	POS	1.07

Figure S1. Phylogenetic tree using HEV RNA positive samples of panel I (N = 15) and reference sequences proposed by Smith D.B. et al. [2] and Muñoz-Chimeno M. et al. [31].

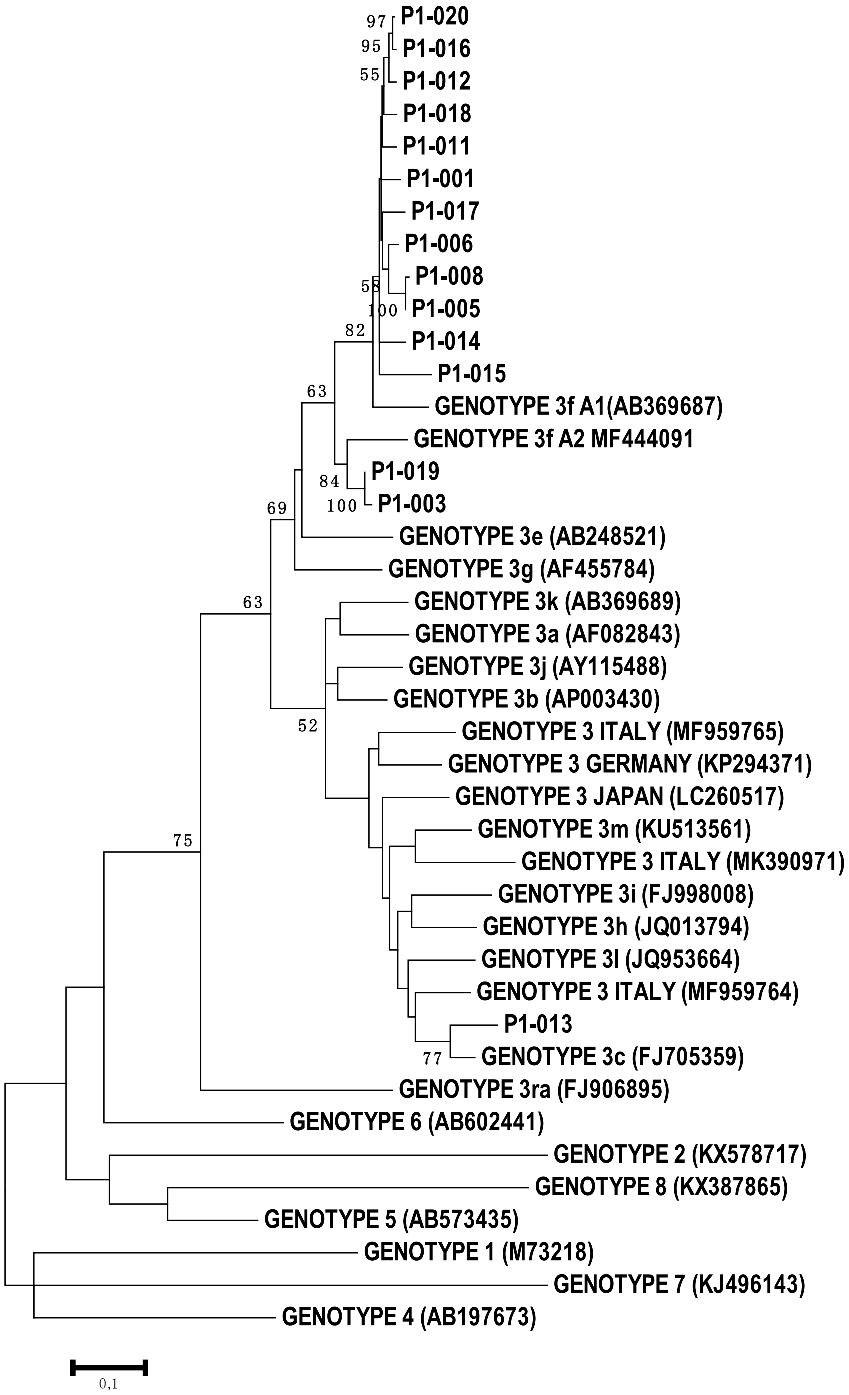

Table S2. Raw data of anti-HEV IgG panel III samples in four used techniques (LIAISON, VIRCLIA, WANTAI and VIDAS). POS: positive result, NEG: negative result.

Panel	Sample ID	LIAISON		VIRCLIA		WANTAI		VIDAS	
		Label	Index	Label	Index	Label	Index	Label	Index
IgG POS (III)	P3-001	POS	0.587	POS	4.534	POS	3.77	POS	1.29
IgG POS (III)	P3-002	POS	>10.0	POS	21.446	POS	12.73	POS	>10.00
IgG POS (III)	P3-003	POS	0.627	POS	4.913	POS	3.32	POS	1.36
IgG POS (III)	P3-004	POS	>10.0	POS	14.028	POS	13.79	POS	>10.00
IgG POS (III)	P3-005	POS	6.85	POS	15.616	POS	14.36	POS	>10.00
IgG POS (III)	P3-006	POS	>10.0	POS	16.759	POS	14.82	POS	>10.00
IgG POS (III)	P3-007	POS	>10.0	POS	22735	POS	15.02	POS	>10.00
IgG POS (III)	P3-008	POS	1.74	POS	9.109	POS	8.44	POS	4.67
IgG POS (III)	P3-009	POS	>10.0	POS	21.309	POS	13.89	POS	>10.00
IgG POS (III)	P3-010	POS	1.44	POS	6.748	POS	7.87	POS	2.87
IgG POS (III)	P3-011	POS	>10.0	POS	15.081	POS	14.62	POS	>10.00
IgG POS (III)	P3-012	POS	>10.0	POS	17.049	POS	15.09	POS	>10.00
IgG POS (III)	P3-013	POS	>10.0	POS	19.23	POS	13.81	POS	>10.00
IgG POS (III)	P3-014	POS	0.966	POS	3.93	POS	3.75	POS	1.29
IgG POS (III)	P3-015	POS	>10.0	POS	16.395	POS	15.23	POS	9.64
IgG POS (III)	P3-016	POS	1.52	POS	8.116	POS	8.87	POS	2.83
IgG POS (III)	P3-017	POS	>10.0	POS	16.849	POS	14.89	POS	>10.00
IgG POS (III)	P3-018	POS	1.13	POS	7.883	POS	7.43	POS	2.59
IgG POS (III)	P3-019	POS	2.93	POS	8.921	POS	11.66	POS	4.4
IgG POS (III)	P3-020	POS	>10.0	POS	15.931	POS	14.23	POS	>10.00
IgG POS (III)	P3-021	POS	3.25	POS	9.247	POS	10.04	POS	4.3
IgG POS (III)	P3-022	POS	0.713	POS	4.608	POS	3.45	POS	1.55
IgG POS (III)	P3-023	POS	0.451	POS	3.065	POS	2.11	POS	0.96
IgG POS (III)	P3-024	POS	1.46	POS	12.251	POS	9.43	POS	1.49
IgG POS (III)	P3-025	POS	0.798	POS	3.343	POS	3.83	POS	1.3
IgG POS (III)	P3-026	POS	>10.0	POS	23.519	POS	13.63	POS	>10.00
IgG POS (III)	P3-027	POS	0.968	POS	4.259	POS	4.52	POS	2.03
IgG POS (III)	P3-028	POS	>10.0	POS	22.978	POS	14.06	POS	>10.00
IgG POS (III)	P3-029	POS	0.812	POS	4.208	POS	4.05	POS	1.8
IgG POS (III)	P3-030	POS	0.436	POS	2.967	POS	2.05	POS	0.86
IgG POS (III)	P3-031	POS	1.94	POS	12.382	POS	7.29	POS	3.7
IgG POS (III)	P3-032	POS	1.52	POS	5.098	POS	7.11	POS	2.17
IgG POS (III)	P3-033	POS	>10.0	POS	19.438	POS	14.95	POS	>10.00
IgG POS (III)	P3-034	POS	2.76	POS	9.651	POS	12.09	POS	4.87
IgG POS (III)	P3-035	POS	>10.0	POS	16.872	POS	13.80	POS	>10.00
IgG POS (III)	P3-036	POS	>10.0	POS	18.383	POS	14.14	POS	>10.00
IgG POS (III)	P3-037	POS	0.91	POS	4.658	POS	3.54	POS	1.82
IgG POS (III)	P3-038	POS	2.4	POS	10.652	POS	8.63	POS	4.72
IgG POS (III)	P3-039	POS	>10.0	POS	10.642	POS	13.99	POS	>10.00
IgG POS (III)	P3-040	POS	>10.0	POS	17.562	POS	12.51	POS	>10.00
IgG POS (III)	P3-041	POS	5.74	POS	12.077	POS	13.65	POS	9.7

IgG POS (III)	P3-042	POS	4.29	POS	11.913	POS	13.89	POS	>10.00
IgG POS (III)	P3-043	POS	>10.0	POS	15.621	POS	14.72	POS	>10.00
IgG POS (III)	P3-044	POS	>10.0	POS	14.733	POS	15.31	POS	>10.00
IgG POS (III)	P3-045	POS	>10.0	POS	15.148	POS	14.36	POS	>10.00
IgG POS (III)	P3-046	POS	>10.0	POS	14.457	POS	13.95	POS	>10.00
IgG POS (III)	P3-047	POS	1.4	POS	6.94	POS	8.04	POS	2.7
IgG POS (III)	P3-048	POS	2.36	POS	9.584	POS	10.11	POS	4.13
IgG POS (III)	P3-049	POS	0.894	POS	4.521	POS	3.37	POS	1.73
IgG POS (III)	P3-050	POS	2.7	POS	9.586	POS	11.83	POS	5.36
IgG POS (III)	P3-051	POS	>10.0	POS	23.624	POS	13.20	POS	>10.00
IgG POS (III)	P3-052	POS	6.39	POS	13.321	POS	12.04	POS	9.3
IgG POS (III)	P3-053	POS	>10.0	POS	27.416	POS	14.68	POS	>10.00
IgG POS (III)	P3-054	POS	8.51	POS	18.086	POS	14.98	POS	>10.00
IgG POS (III)	P3-055	POS	>10.0	POS	25.879	POS	14.17	POS	>10.00
IgG POS (III)	P3-056	POS	>10.0	POS	16.811	POS	14.52	POS	>10.00
IgG POS (III)	P3-057	POS	>10.0	POS	17.861	POS	14.45	POS	>10.00
IgG POS (III)	P3-058	POS	>10.0	POS	24.022	POS	13.73	POS	>10.00
IgG POS (III)	P3-059	POS	>10.0	POS	21.892	POS	14.30	POS	>10.00
IgG POS (III)	P3-060	POS	2.05	POS	9.022	POS	8.83	POS	4.62
IgG POS (III)	P3-061	POS	0.303	POS	1.427	NEG	0.62	POS	0.66
IgG POS (III)	P3-062	POS	3.26	POS	9.41	POS	12.82	POS	5.56
IgG POS (III)	P3-063	POS	7.8	POS	13.884	POS	14.68	POS	>10.00
IgG POS (III)	P3-064	POS	5.05	POS	14.27	POS	14.64	POS	8.35
IgG POS (III)	P3-065	POS	>10.0	POS	14.352	POS	13.71	POS	>10.00
IgG POS (III)	P3-066	POS	0.371	POS	2.35	POS	1.44	POS	0.72
IgG POS (III)	P3-067	POS	2.45	POS	12.391	POS	13.16	POS	7.25
IgG POS (III)	P3-068	POS	>10.0	POS	19.907	POS	14.37	POS	>10.00
IgG POS (III)	P3-069	POS	2.08	POS	5.337	POS	8.98	POS	2.72
IgG POS (III)	P3-070	POS	5.1	POS	12.122	POS	14.63	POS	9.26
IgG POS (III)	P3-071	POS	>10.0	POS	17.65	POS	15.69	POS	>10.00
IgG POS (III)	P3-072	POS	>10.0	POS	19.862	POS	12.65	POS	>10.00
IgG POS (III)	P3-073	POS	2.97	POS	8.384	POS	7.62	POS	5.19
IgG POS (III)	P3-074	POS	0.96	POS	3.548	POS	3.61	POS	1.56
IgG POS (III)	P3-075	POS	0.39	POS	2.392	POS	1.37	POS	0.75
IgG POS (III)	P3-076	POS	>10	POS	26.264	POS	16.20	POS	>10.00
IgG POS (III)	P3-077	POS	0.778	POS	8.095	POS	4.26	POS	1.85
IgG POS (III)	P3-078	POS	>10	POS	32.689	POS	14.60	POS	>10.00
IgG POS (III)	P3-079	POS	>10	POS	28.508	POS	14.88	POS	>10.00
IgG POS (III)	P3-080	POS	3.72	POS	14.574	POS	10.59	POS	5.23
IgG POS (III)	P3-081	POS	3.47	POS	16.813	POS	13.93	POS	7.89
IgG POS (III)	P3-082	POS	1.84	POS	17.21	POS	6.25	POS	4.34
IgG POS (III)	P3-083	POS	7.97	POS	18.732	POS	10.97	POS	9.69
IgG POS (III)	P3-084	POS	0.698	POS	5.805	POS	1.74	POS	1.64
IgG POS (III)	P3-085	POS	8.24	POS	20.537	POS	15.92	POS	>10.00
IgG POS (III)	P3-086	POS	2.69	POS	10.673	POS	8.23	POS	2.84
IgG POS (III)	P3-087	POS	2	POS	10.685	POS	9.71	POS	3.98

IgG POS (III)	P3-088	POS	>10	POS	24.788	POS	16.49	POS	>10.00
IgG POS (III)	P3-089	POS	1.07	POS	5.042	POS	2.87	POS	1.99
IgG POS (III)	P3-090	POS	>10	POS	23.658	POS	17.58	POS	>10.00
IgG POS (III)	P3-091	POS	1.56	POS	8.652	POS	8.86	POS	3.07
IgG POS (III)	P3-092	POS	>10	POS	19.709	POS	15.85	POS	>10.00
IgG POS (III)	P3-093	POS	>10	POS	22.73	POS	16.00	POS	>10.00
IgG POS (III)	P3-094	POS	2.5	POS	9.874	POS	12.92	POS	4.00
IgG POS (III)	P3-095	POS	1.22	POS	6.424	POS	7.59	POS	1.78
IgG POS (III)	P3-096	POS	4.34	POS	16.542	POS	16.08	POS	9.6
IgG POS (III)	P3-097	POS	2	POS	9.503	POS	8.77	POS	2.74
IgG POS (III)	P3-098	POS	>10	POS	23.879	POS	16.63	POS	>10.00
IgG POS (III)	P3-099	POS	0.459	POS	4.145	POS	1.43	POS	1.12
IgG POS (III)	P3-100	POS	>10	POS	23.13	POS	16.38	POS	>10.00

Table S3. Raw data of negative panels IV and V in both LIAISON and VIRCLIA techniques. POS: positive result, NEG: negative result.

Panel	Sample ID	anti-HEV IgM				anti-HEV IgG			
		LIAISON		VIRCLIA		LIAISON		VIRCLIA	
		Label	Index	Label	Index	Label	Index	Label	Index
NEG (IV-V)	PN-001	NEG	<0.100	NEG	0,02	NEG	<0.100	POS	1,426
NEG (IV-V)	PN-002	NEG	<0.100	NEG	0,026	NEG	<0.100	NEG	0,285
NEG (IV-V)	PN-003	NEG	<0.100	NEG	0,053	NEG	0.190	POS	1,8
NEG (IV-V)	PN-004	NEG	<0.100	NEG	0,023	NEG	<0.100	NEG	0,212
NEG (IV-V)	PN-005	NEG	0.134	NEG	0,063	NEG	<0.100	NEG	0,284
NEG (IV-V)	PN-006	NEG	<0.100	NEG	0,026	NEG	<0.100	NEG	0,355
NEG (IV-V)	PN-007	NEG	<0.100	NEG	0,035	NEG	<0.100	NEG	0,3
IgM NEG (IV)	PN-008	NEG	<0.100	NEG	0,026				
NEG (IV-V)	PN-009	NEG	<0.100	NEG	0,023	NEG	<0.100	NEG	0,274
NEG (IV-V)	PN-010	NEG	<0.100	NEG	0,026	NEG	<0.100	NEG	0,479
NEG (IV-V)	PN-011	NEG	0.111	NEG	0,026	NEG	0.238	POS	2,451
NEG (IV-V)	PN-012	NEG	<0.100	NEG	0,025	NEG	<0.100	NEG	0,475
NEG (IV-V)	PN-013	NEG	<0.100	NEG	0,038	NEG	<0.100	NEG	0,343
IgM NEG (IV)	PN-014	NEG	0.747	NEG	0,033				
NEG (IV-V)	PN-015	NEG	<0.100	NEG	0,039	NEG	0.140	NEG	0,378
NEG (IV-V)	PN-016	NEG	<0.100	NEG	0,026	NEG	<0.100	NEG	0,31
NEG (IV-V)	PN-017	NEG	0.113	NEG	0,026	NEG	<0.100	NEG	0,4
NEG (IV-V)	PN-018	NEG	0.269	NEG	0,042	NEG	0,696	NEG	0,83
NEG (IV-V)	PN-019	NEG	<0.100	NEG	0,029	NEG	<0.100	NEG	0,388
NEG (IV-V)	PN-020	NEG	<0.100	NEG	0,027	NEG	<0.100	NEG	0,469
NEG (IV-V)	PN-021	NEG	<0.100	NEG	0,046	NEG	<0.100	NEG	0,433
NEG (IV-V)	PN-022	NEG	<0.100	NEG	0,027	NEG	<0.100	NEG	0,363

NEG (IV-V)	PN-023	NEG	<0.100	NEG	0,026	NEG	<0.100	NEG	0,233
NEG (IV-V)	PN-024	NEG	<0.100	NEG	0,028	NEG	<0.100	IND	1,099
NEG (IV-V)	PN-025	NEG	<0.100	NEG	0,031	NEG	<0.100	NEG	0,312
NEG (IV-V)	PN-026	NEG	<0.100	NEG	0,026	NEG	<0.100	NEG	0,16
NEG (IV-V)	PN-027	NEG	<0.100	NEG	0,049	NEG	<0.100	NEG	0,415
IgM NEG (IV)	PN-028	NEG	<0.100	NEG	0,036				
NEG (IV-V)	PN-029	NEG	<0.100	NEG	0,031	NEG	<0.100	NEG	0,278
IgM NEG (IV)	PN-030	NEG	<0.100	NEG	0,026				
NEG (IV-V)	PN-031	NEG	<0.100	NEG	0,054	NEG	0.115	NEG	0,491
IgM NEG (IV)	PN-032	NEG	<0.100	NEG	0,034				
NEG (IV-V)	PN-033	NEG	<0.100	NEG	0,028	NEG	<0.100	NEG	0,477
IgM NEG (IV)	PN-034	NEG	0.171	NEG	0,048				
NEG (IV-V)	PN-035	NEG	<0.100	NEG	0,027	NEG	<0.100	NEG	0,366
NEG (IV-V)	PN-036	NEG	<0.100	NEG	0,026	NEG	<0.100	NEG	0,259
NEG (IV-V)	PN-037	NEG	<0.100	NEG	0,039	NEG	<0.100	POS	1,52
NEG (IV-V)	PN-038	NEG	0.130	NEG	0,038	NEG	<0.100	NEG	0,324
NEG (IV-V)	PN-039	NEG	<0.100	NEG	0,042	NEG	0.120	POS	1,507
NEG (IV-V)	PN-040	NEG	<0.100	NEG	0,028	NEG	<0.100	NEG	0,252
IgM NEG (IV)	PN-041	NEG	0.288	NEG	0,04				
IgM NEG (IV)	PN-042	NEG	<0.100	NEG	0,035				
NEG (IV-V)	PN-043	NEG	<0.100	NEG	0,03	NEG	<0.100	NEG	0,351
NEG (IV-V)	PN-044	NEG	<0.100	NEG	0,031	NEG	<0.100	NEG	0,415
NEG (IV-V)	PN-045	NEG	<0.100	NEG	0,033	NEG	<0.100	NEG	0,706
NEG (IV-V)	PN-046	NEG	<0.100	NEG	0,031	NEG	0.137	NEG	0,7
NEG (IV-V)	PN-047	NEG	<0.100	NEG	0,035	NEG	0.220	NEG	0,434
NEG (IV-V)	PN-048	NEG	<0.100	NEG	0,036	NEG	<0.100	NEG	0,236
NEG (IV-V)	PN-049	NEG	<0.100	NEG	0,028	NEG	<0.100	NEG	0,368
NEG (IV-V)	PN-050	NEG	<0.100	NEG	0,032	NEG	<0.100	NEG	0,753
NEG (IV-V)	PN-051	NEG	0.361	NEG	0,034	NEG	<0.100	NEG	0,294
NEG (IV-V)	PN-052	NEG	0.185	NEG	0,038	NEG	0.196	NEG	0,762
IgM NEG (IV)	PN-053	NEG	0.150	NEG	0,089				
NEG (IV-V)	PN-054	NEG	<0.100	NEG	0,034	NEG	<0.100	NEG	0,44
NEG (IV-V)	PN-055	NEG	<0.100	NEG	0,028	NEG	<0.100	NEG	0,363
NEG (IV-V)	PN-056	NEG	0.532	NEG	0,035	NEG	<0.100	NEG	0,382
NEG (IV-V)	PN-057	NEG	<0.100	NEG	0,025	NEG	<0.100	NEG	0,822
NEG (IV-V)	PN-058	NEG	<0.100	NEG	0,029	NEG	<0.100	POS	3,886
NEG (IV-V)	PN-059	NEG	<0.100	NEG	0,027	NEG	<0.100	NEG	0,224
IgM NEG (IV)	PN-060	NEG	<0.100	NEG	0,023				
NEG (IV-V)	PN-061	NEG	0.172	NEG	0,028	NEG	<0.100	NEG	0,229
NEG (IV-V)	PN-062	NEG	<0.100	NEG	0,026	POS	0,373	POS	2,246
NEG (IV-V)	PN-063	NEG	<0.100	NEG	0,031	NEG	<0.100	NEG	0,218

NEG (IV-V)	PN-064	NEG	0.424	NEG	0,024	NEG	<0.100	NEG	0,219
NEG (IV-V)	PN-065	NEG	<0.100	NEG	0,027				
NEG (IV-V)	PN-066	NEG	0.196	NEG	0,035	NEG	<0.100	NEG	0,2
NEG (IV-V)	PN-067	NEG	0.122	NEG	0,055				
NEG (IV-V)	PN-068	NEG	<0.100	NEG	0,025	NEG	<0.100	NEG	0,149
NEG (IV-V)	PN-069	NEG	0.132	NEG	0,047	NEG	<0.100	NEG	0,619
NEG (IV-V)	PN-070	NEG	0.186	NEG	0,028	NEG	<0.100	NEG	0,058
NEG (IV-V)	PN-071	NEG	<0.100	NEG	0,016	NEG	<0.100	NEG	0,205
NEG (IV-V)	PN-072	NEG	<0.100	NEG	0,035	NEG	<0.100	NEG	0,127
NEG (IV-V)	PN-073	NEG	<0.100	NEG	0,038	NEG	<0.100	NEG	0,388
NEG (IV-V)	PN-074	NEG	0.228	NEG	0,027	NEG	0.184	POS	1,568
NEG (IV-V)	PN-075	NEG	<0.100	NEG	0,03	NEG	<0.100	NEG	0,29
NEG (IV-V)	PN-076	NEG	0.198	NEG	0,023	NEG	<0.100	NEG	0,189
NEG (IV-V)	PN-077	NEG	<0.100	NEG	0,019	NEG	<0.100	NEG	0,247
NEG (IV-V)	PN-078	NEG	<0.100	NEG	0,026	NEG	<0.100	NEG	0,197
NEG (IV-V)	PN-079	NEG	<0.100	NEG	0,133	NEG	<0.100	NEG	0,285
NEG (IV-V)	PN-080	NEG	<0.100	NEG	0,02	NEG	<0.100	NEG	0,702
NEG (IV-V)	PN-081	NEG	<0.100	NEG	0,019	NEG	<0.100	NEG	0,379
NEG (IV-V)	PN-082	NEG	0.294	NEG	0,021	NEG	<0.100	NEG	0,235
NEG (IV-V)	PN-083	NEG	<0.100	NEG	0,033	NEG	<0.100	NEG	0,711
NEG (IV-V)	PN-084	NEG	0.154	NEG	0,032	NEG	<0.100	NEG	0,129
NEG (IV-V)	PN-085	NEG	<0.100	NEG	0,017	NEG	<0.100	NEG	0,125
NEG (IV-V)	PN-086	NEG	0.874	NEG	0,017	NEG	0.129	NEG	0,254
NEG (IV-V)	PN-087	NEG	<0.100	NEG	0,027	NEG	<0.100	NEG	0,271
NEG (IV-V)	PN-088	NEG	<0.100	NEG	0,029	POS	0,35	POS	1,335
IgM NEG (IV)	PN-089	NEG	0.182	NEG	0,023				
NEG (IV-V)	PN-090	NEG	<0.100	NEG	0,021	NEG	<0.100	NEG	0,19
NEG (IV-V)	PN-091	NEG	0.214	NEG	0,03	NEG	<0.100	NEG	0,295
NEG (IV-V)	PN-092	NEG	0.303	NEG	0,023	NEG	<0.100	NEG	0,344
NEG (IV-V)	PN-093	NEG	0.396	NEG	0,018	NEG	<0.100	POS	1,547
NEG (IV-V)	PN-094	NEG	<0.100	NEG	0,021	NEG	<0.100	NEG	0,098
NEG (IV-V)	PN-095	NEG	0.114	NEG	0,022	NEG	<0.100	NEG	0,151
NEG (IV-V)	PN-096	NEG	0.155	NEG	0,034	NEG	<0.100	NEG	0,272
IgM NEG (IV)	PN-097	NEG	0.126	NEG	0,173				
NEG (IV-V)	PN-098	NEG	<0.100	NEG	0,033	NEG	<0.100	NEG	0,342
NEG (IV-V)	PN-099	NEG	<0.100	NEG	0,032	NEG	<0.100	NEG	0,286
NEG (IV-V)	PN-100	NEG	<0.100	NEG	0,03	NEG	<0.100	NEG	0,277
NEG (IV-V)	PN-101	NEG	0.141	NEG	0,031	NEG	<0.100	NEG	0,707
IgG NEG (V)	PN-102					NEG	<0.100	NEG	0,357
IgG NEG (V)	PN-103					NEG	<0.100	NEG	0,173
IgG NEG (V)	PN-104					NEG	<0.100	NEG	0,172

IgG NEG (V)	PN-105		NEG	<0.100	NEG	0,248
IgG NEG (V)	PN-106		NEG	<0.100	NEG	0,219
IgG NEG (V)	PN-107		NEG	<0.100	NEG	0,238
IgG NEG (V)	PN-108		NEG	<0.100	NEG	0,278
IgG NEG (V)	PN-109		NEG	<0.100	NEG	0,251
IgG NEG (V)	PN-110		NEG	<0.100	NEG	0,29
IgG NEG (V)	PN-111		NEG	<0.100	NEG	0,274
IgG NEG (V)	PN-112		NEG	<0.100	NEG	0,256
IgG NEG (V)	PN-113		NEG	<0.100	NEG	0,223
IgG NEG (V)	PN-114		NEG	<0.100	NEG	0,337
IgG NEG (V)	PN-115		NEG	<0.100	NEG	0,214
IgG NEG (V)	PN-116		NEG	<0.100	NEG	0,604
IgG NEG (V)	PN-117		NEG	<0.100	NEG	0,227
IgG NEG (V)	PN-118		NEG	<0.100	NEG	0,246
IgG NEG (V)	PN-119		NEG	<0.100	NEG	0,277
IgG NEG (V)	PN-120		NEG	0,183	IND	1,042
